# Generation of cardiomyocytes from human-induced pluripotent stem cells resembling atrial cells with ability to respond to adrenoceptor agonists

**DOI:** 10.1098/rstb.2022.0312

**Published:** 2023-06-19

**Authors:** Faizzan S. Ahmad, Yongcheng Jin, Alexander Grassam-Rowe, Yafei Zhou, Meng Yuan, Xuehui Fan, Rui Zhou, Razik Mu-u-min, Christopher O'Shea, Ayman M. Ibrahim, Wajiha Hyder, Yasmine Aguib, Magdi Yacoub, Davor Pavlovic, Yanmin Zhang, Xiaoqiu Tan, Ming Lei, Derek A. Terrar

**Affiliations:** ^1^ Department of Pharmacology, University of Oxford, Mansfield Road, Oxford OX1 3QT, UK; ^2^ Cure8bio, Inc, 395 Fulton Street, Westbury, NY 11590, USA; ^3^ Key Laboratory of Medical Electrophysiology of the Ministry of Education and Institute of Cardiovascular Research, Southwest Medical University, Luzhou 6400, People's Republic of China; ^4^ Shaanxi Institute for Pediatric Diseases, Department of Cardiology, Xi'an Children's Hospital, Xi'an 710003, People's Republic of China; ^5^ Lombardi Comprehensive Cancer Center, Georgetown University, Washington, DC 20057, USA; ^6^ Institute of Cardiovascular Sciences, College of Medicine and Dental Sciences, University of Birmingham, Birmingham B15 2TT, UK; ^7^ Aswan Heart Centre, Aswan 1242770, Egypt; ^8^ Department of Zoology, Faculty of Science, Cairo University, Cairo 12613, Egypt; ^9^ National Heart and Lung Institute, Heart Science Centre, Imperial College London, Middlesex SW3 6LY, UK

**Keywords:** iPSCs, iPSC-derived atrial cardiomyocytes, Gremlin 2, retinoic acid, adrenergic receptor, calcium

## Abstract

Atrial fibrillation (AF) is the most common chronic arrhythmia presenting a heavy disease burden. We report a new approach for generating cardiomyocytes (CMs) resembling atrial cells from human-induced pluripotent stem cells (hiPSCs) using a combination of Gremlin 2 and retinoic acid treatment. More than 40% of myocytes showed rod-shaped morphology, expression of CM proteins (including ryanodine receptor 2, *α*-actinin-2 and F-actin) and striated appearance, all of which were broadly similar to the characteristics of adult atrial myocytes (AMs). Isolated myocytes were electrically quiescent until stimulated to fire action potentials with an AM profile and an amplitude of approximately 100 mV, arising from a resting potential of approximately −70 mV. Single-cell RNA sequence analysis showed a high level of expression of several atrial-specific transcripts including *NPPA*, *MYL7*, *HOXA3*, *SLN*, *KCNJ4*, *KCNJ5* and *KCNA5*. Amplitudes of calcium transients recorded from spontaneously beating cultures were increased by the stimulation of *α*-adrenoceptors (activated by phenylephrine and blocked by prazosin) or *β*-adrenoceptors (activated by isoproterenol and blocked by CGP20712A). Our new approach provides human AMs with mature characteristics from hiPSCs which will facilitate drug discovery by enabling the study of human atrial cell signalling pathways and AF.

This article is part of the theme issue ‘The heartbeat: its molecular basis and physiological mechanisms’.

## 1. Introduction

Atrial fibrillation (AF) is the most frequently encountered arrhythmia in clinical practice and represents a significant disease burden worldwide, with a high prevalence and ability to cause morbidity and mortality in the population, particularly in the elderly [1]. Current treatments of AF have major limitations including limited efficacy and significant adverse effect liability. These limitations have inspired substantial efforts concerning mechanistic research and innovative approaches for the development of new therapies, such as tailoring treatment to the underlying pathophysiology of AF [2]. For achieving such a goal, suitable model systems replicating the adult human atrial cardiomyocyte (CM) phenotype are required.

In recent years, human-induced pluripotent stem cells (iPSCs) have emerged as an alternative *in vitro* model system to the use of animals for investigation of human disease mechanisms and development of new medications. Numerous disease-specific iPSC lines have been produced for modelling congenital cardiac arrhythmia syndromes, including heritable AF [3–5]. Although iPSCs hold great promise for heart disease research and treatment, there are obvious obstacles to be overcome. For instance, cardiogenic differentiation of iPSCs by existing protocols often produces a heterogeneous mixture of CMs of different subtypes, primarily ventricular CMs. More importantly, a major problem is the maturation of CMs from hiPSCs, since the resultant cells frequently display developmentally immature characteristics which are analogous to fetal CMs [6,7]. The immaturity of CMs derived from hiPSCs makes them less suitable models for studying the most common heart diseases, that typically occur in adulthood, and also diminishes the suitability of such models for drug screening. Over the past few years, great efforts have been made by a number of groups in developing new approaches in the generation of human embryonic stem cell (hESC)- or hiPSC-derived atrial CMs [8–15]. A particular molecule that has attracted attention and is being used for generating hESC- or hiPSC-derived atrial CMs is retinoic acid (RA), a metabolite of vitamin A1, that mediates the functions of vitamin A1 required for growth and development. An earlier elegant study reported by Keller's group [16] determined RA and WNT signalling as key regulators of human stem cell development. Their study thus provides molecular insights that aid in developing strategies for the generation of atrial CMs from PSCs. Additionally, other groups have inspected Gremlin2 as a pro-atrial differentiation factor and reported promising results [17]. Gremlin 2 is a signalling molecule involved in cardiac development and atrial-specific differentiation as cardiac progenitor cells migrate [17–19]. These studies have thus prompted the efforts in developing protocols for generating hESC- or hiPSC-derived atrial CMs. Atrial CMs derived by these different approaches showed enhanced expression of atrium-specific genes and reduced expression of ventricle-specific genes, but, these cells still show important differences from mature atrial myocytes (AMs; table 1), particularly in their electrophysiological characteristics: for example, such cells frequently lack a stable resting potential, or when there is a stable resting potential the level is substantially more positive than that observed in the adult phenotype.

Here we report a new approach that is able to generate hiPSC-derived atrial myocytes (hiPSC-AMs) showing mature characteristics, using a newly developed differentiation protocol involving a treatment regimen combining RA and Gremlin 2. With our RA- and Gremlin 2-based differentiation protocol, we observed a high proportion of elongated cells, some of which showed a remarkable adult AM-like morphology. Action potentials (APs) with an atrial-type morphology were recorded following electrical stimulation of quiescent cells, and the cells also showed a remarkable response to adrenergic stimulation that has not been shown in previous reports in RA-guided differentiation protocols, to the authors' knowledge [11–13]. CMs differentiated from hiPSCs using our new RA/Gremlin 2 protocol also showed an atrial-like transcriptomic profile with high level of expression of several atrial-specific transcripts including *NPPA* (natriuretic peptide A), *MYL7* (myosin light chain 2, atrial isoform), *KCNJ5* (Kir3.4 subunit for conducting I_KACh_) and *KCNA5* (K_V_1.5 for conducting I_Kur_) as compared with a control group treated with a standard conventional commercial CM differentiation protocol. Our new method therefore provides an effective approach for differentiating human AMs with mature characteristics from iPSCs. These cells are likely to be very useful for studying the presence and function of components of cell signalling pathways in human AMs and will probably provide a valuable model for modelling AF disease and drug discovery.

## 2. Results

### (a) Morphological and immunocytochemistry characterization reveals a more mature atrial-like structure in Gremlin 2/retinoic acid-treated cells

**Figure 1.**
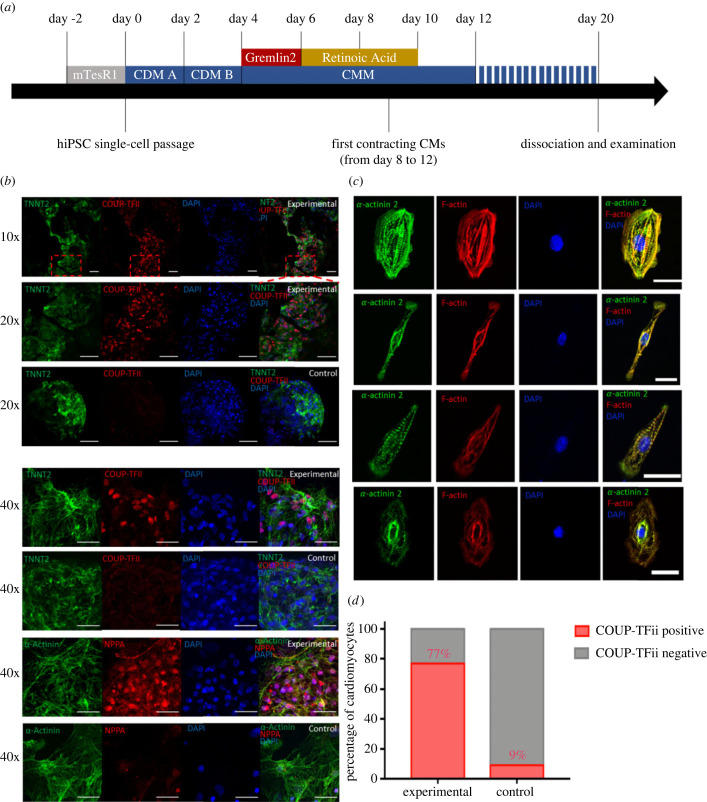
Gremlin2/RA-mediated differentiation of iPSCs into atrial cardiomyocytes (CMs). (*a*) Schematic of the atrial-specific differentiation protocols. Human iPSCs were dissociated into single CMs for the differentiation experiments. mTeSR1 is a defined tissue culture medium from Stemcell Technologies. Cardiac differentiation medium A (CDM A) including CHIR99021 and cardiac differentiation medium B (CDM B) were applied to both groups. From day 4, all CMs were maintained in CMM. In the Experimental group, Gremlin 2 (1 mg ml^−1^) was added at day 4, and of RA (1.0 mM) was added with Gremlin 2 still present at day 6, while the control group of cells was differentiated without the addition of Gremlin 2 and RA. (*b*) Functional analysis was performed after day 20 when both cell populations of hiPSC-derived CMs showed the expression of the CM-specific cytoskeleton markers *α*-actinin 2 (ACTN2) and TNNT2 (cardiac troponin C2), while the expression of the atrial CM-specific markers COUP-TFII and NPPA was restricted to the experimental group. (*c*) Immunofluorescence labelling of the cytoskeleton markers *α*-actinin 2 and F-actin for cells in the experimental group demonstrating an elongated morphology with striations. (*d*) The percentage of COUP-TFII positive CMs was 77% in the experimental Gremlin2/RA-treated group. A total of approximately 1000 DAPI-stained cell nuclear were identified in both treated and control groups, and then they were overlapped with COUP-TFII positive nuclear to calculate the percentage of COUP-TFII positive cells. A Chi-square test was applied, *n* = 1000, *p* < 0.001.

Previous studies showed that the *in vitro* specification of CM subtypes is initiated after the induction of mesoderm and before the terminal differentiation of cardiac progenitor cells [9,12]. Therefore, we speculated that a combination of the Gremlin 2 and RA treatment within this time window might be more effective for directing differentiation of iPSCs towards a cardiac atrial phenotype. Thus, we developed our atrial-specific differentiation protocol as shown by the schematic diagram in figure 1*a*, with the detailed protocol described in Appendix S1. Briefly, in this protocol, Gremlin 2 was added prior to RA. We then characterized the functional and structural properties of the resulting derived cells, as described below. The experiments were conducted from two independent iPSC lines and multiple batches of cells.

The cells prepared from the control group (commercial PSC Cardiac Differentiation Kit Gibco™ Technologies, cat. no. A2921201) and cells treated with Gremlin 2/RA (defined as the experimental group) were characterized by immunocytochemistry to determine their morphology and expression of CM-specific proteins, particularly those expressed specifically in AMs. The cytoskeletal protein ACTN2 (*α*-actinin 2) and TNNT2 (cardiac troponin C2) were in both control and experimental (day 20) cell cultures (figure 1*b*). However, labelling for atrial-specific NPPA (natriuretic peptide A) and COUP-TFII (also known as nuclear family receptor 2, group F, member 2) was clearly evident in the experimental group but not control group (figure 1*b*). Further immunohistochemistry was carried out in individual cells following single-cell isolation from cell cultures (see Material and Methods). We observed the same general expression of ACTN2 and TNNT2 in both experimental and control isolated CM groups, but only NPPA and COUP-TFII expression in the experimental Gremlin 2/RA-treated group (figure 1*c*).

The morphology of some of iPSC-derived AMs shown in figure 1*c* was remarkably similar to that of adult AMs observed in human and animal hearts [15,35]. An elongated rod-like morphology resembling the adult phenotype was observed across much of our experimental group (figure 1*c*), which was comparable to that reported in human mature AMs [15,35]. Furthermore, our Gremlin2/RA experimental group also showed a well-organized striated sarcomeric pattern with sarcomere spacing slightly less than 2 mm (figure 1*c*), as observed in adult CMs [15,35]. The percentage of CMs in the experimental group that were positive for the atrial-specific COUP-TFII/NPPA was 77% from approximately 1000 counted cells, in contrast with less than 10% in the control group (figure 1*d*); thus supporting that our hiPSC-AM differentiation protocol established robust protein expression for selected major atrial transcription factors and associated atrial markers.

### (b) Transcriptional characterization identifies a more robust atrial transcriptional program in Gremlin 2/retinoic acid-treated cells

**Figure 2.**
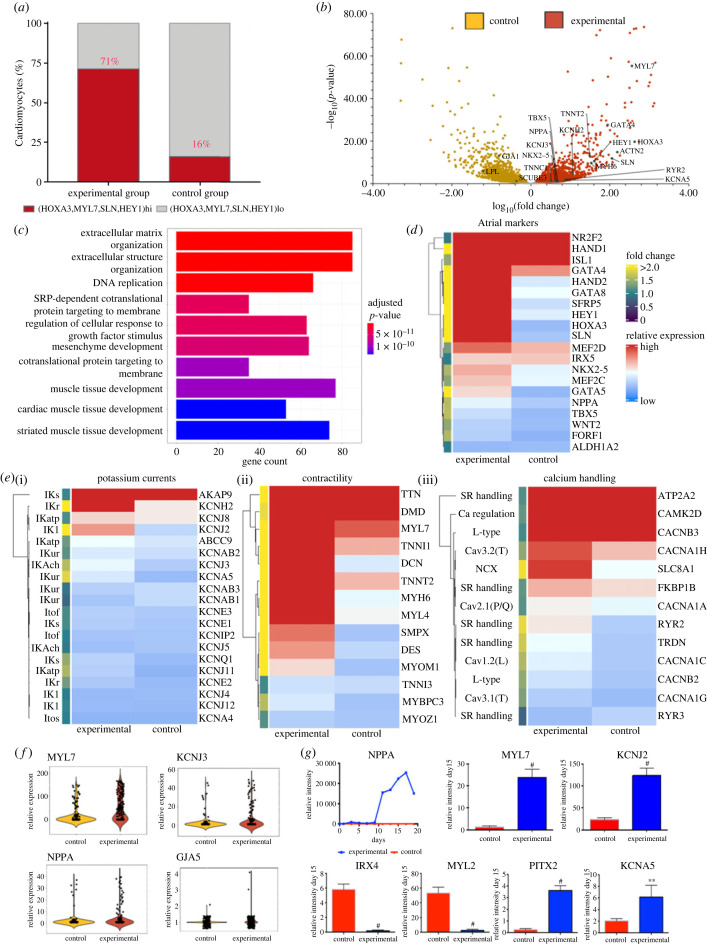
Single-cell transcriptomic profile. (*a*) Plot showing the percentage of cardiomyocytes (CMs) expressing atrial-specific markers HEY1, MYL7, HOXA3 and SLN in the experimental and control groups. (*b*) Differential gene expression analysis showed many upregulated atrial-specific gene in experimental group with selected markers indicated. (*c*) Gene ontology (GO) plot showing the top 10 most enriched GO terms in the genes differentially expressed between the control and experimental group. Coloured by Bonferroni–Hochberg-adjusted *p*-value. Gene count along the *x*-axis marks the number of genes enriched within each GO term. (*d*) Heatmap plot showing the expression of cardiogenic and chamber-specific markers. Cells are coloured by relative expression. The annotation on the left indicates the specific fold change in expression from the control group to the experimental group. (*e*) (i) Heatmap plot showing the expression of genes coding for potassium handling proteins. Cells are coloured by relative expression. The annotation on the left indicates the specific fold change in expression from the control group to the experimental group. The associated currents for each channel subunit are annotated on the left. (ii) Heatmap plot showing the expression of genes coding for contractility-associated proteins. Cells are coloured by relative expression. The annotation on the left indicates the specific fold change in expression from the control group to the experimental group. (iii) Heatmap plot showing the expression of genes coding for calcium-handling proteins. Cells are coloured by relative expression. The annotation on the left indicates the specific fold change in expression from the control group to the experimental group. The associated currents/roles for each protein subunit are annotated on the left. (*f*) Violin plots of individual expression of genes coding for atrial proteins (*MYL7*, *NPPA*, *KCNJ3, GJA5*) at day 20. (*g*) Time-lapse quantitative reverse transcription-polymerase chain reaction (qRT-PCR) gene analysis of *NPPA* over a 20-day time course, and qRT-PCR analysis of expression on day 15 of genes coding for proteins that are specific for either of atrial (*MYL7, KCNJ2, PITX2, KCNA5*) or ventricular (*IRX4, MYL2*) CMs. Values shown are relative to housekeeping gene coding for *HPRT*. *n* = 5 batches of cells. Bars show s.d. of mean. ^#^ indicates *p* < 0.001, and ** indicates *p* < 0.05.

To gain insights into the molecular signature of the iPSC-derived atrial CMs from the experimental group and their differences from the control iPSC-derived CMs, we performed single-cell RNA-sequencing (scRNASeq) on over 300 cells from each control and treatment group. We first sought to characterize the percentage of atrial CMs detected in our sample across an orthogonal gene set to *NR2F2* [36] as in figure 1. The experimental group showed a high expression of four markers that are characteristic of atrial CMs: *HEY1*, *MYL7*, *HOXA3* and *SLN* (figure 2*a*)*.* The experimental group was composed 71% of these atrial CMs, whereas the control group only 16% (figure 2*a*). Thus, our transcriptomic calculation of atrial CM abundance supports our earlier results with proteomic identification and calculation.

To better understand the differences between our experimental and control groups, we identified the differentially expressed genes between the groups (figure 2*b*). Gene expression consistent with atrial CMs (including *MLY7, HEY1, HOXA11, SLN, MYH6, KCNH2, GATA4* and *NPPA*) was upregulated in the experimental group, while iPSC-derived CMs from the control group showed higher expression of certain ventricular CM-specific genes (including *GJA1* coding for CX43*, LPL* coding for lipoprotein lipase and *SCUBE3* coding for signal peptide CUB-EG-containing protein 3) (figure 2*b*). Notably, we found that genes encoding potassium channels K_V_1.5 (*KCNA5*) and K_V_11.1 (*KCNH2)* displayed significantly higher expression in CMs from the experimental group compared with CMs from the control group. These potassium channels contribute significantly to the shape and function of APs in human AMs. *KCNA5* encodes a subunit of a delayed rectifier potassium channel K_V_1.5, underlying the ultra-rapidly activating delayed rectifier K^+^ current *I*_Kur_ found in human atrial CMs [37].

We next sought to further investigate the differentially expressed genes between the two groups beyond the level of isolated genes. We used gene ontology (GO) analysis to ascertain if the differentially expressed genes had any enrichment for shared gene function annotations. We identified the top 10 most enriched GO terms, which suggest a difference between groups across a range of significantly enriched functional annotations, relating to organization of the extracellular matrix, replication and cardiac muscle tissue development (figure 2*c*). Thus, our analysis suggests that our experimental group expresses a diversity of genes of known atrial significance, and that this expression is not just across a few genes but across sets of these genes.

We then sought to identify the expression of specific genes of interest across a range of important modules associated with atrial differentiation and maturation: atrial transcriptional programme markers, potassium channels, contractility and calcium handling. We observe that the experimental group had higher expression of key atrial markers such as *NR2F2, SLN, HEY1, HOXA3* and *NPPA* (figure 2*d*); whereas the control group expressed more ventricular markers, such as *IRX5* that is an important driver in development of the ventricular transcriptional programme [38] (figure 2*d*). However, we note that the experimental group also had increased expression across other genes pertinent for the specification of chamber-specific lineages (figure 2*d*).

Potassium currents are particularly important for defining the specific AP morphology and electrophysiological behaviour of atrial CMs [39]. We saw an increased expression of several potassium channel-encoding genes (figure 2*e*(i)). Of particular interest is the higher expression of *KCNJ2*, that encodes *K*_ir2.1_, which gives rise to the inwardly rectifying I_K1_ current—a current that demarks more mature atrial CMs [40].

Appropriate contractile effort in CMs derives from mature molecular organization of actomyosin filaments into sarcomeres connected through to the cell surface and extracellular matrix. Our experimental group has much higher expression of atrial-specific contractile genes, such as *MYH6, MYL7* and *MYL4*; more general sarcomeric genes, such as *TTN*, *TNNT2, MYOM1* and *MYBPC3*; genes that integrate the sarcomeric contraction with the rest of the cytoskeleton and sarcolemma, such as *DMD* and *DES*; and hypertrophy-associated genes, such as *DCN* and *SMPX* (figure 2*e*(ii)).

Normal calcium handling is of major importance for normal CM function and the use of hiPSC-derived atrial CMs for studying atrial disease. Our data reveals that our experimental group has higher expression of genes coding for proteins associated with both sarcoplasmic reticulum (SR)-associated calcium handling and membrane calcium handling (figure 2*e*(iii)). In particular, our experimental group has higher expression of genes coding for the major SR calcium-handling proteins *RYR2, TRDN and FKBP1B* alongside high expression of the gene coding for *ATP2A2* (figure 2*e*(iii)): whereas the control group had higher expression genes coding for the pre-dominantly non-cardiac *RYR3* isoform [41]*.* Additionally, our experimental group has higher expression of the gene coding for *SLC8A1,* which is a key protein for CM ionic homeostasis, and higher expression of genes coding for T-type calcium channel proteins, *CACNA1H* and *CACNA1G* which contribute to atrial-specific electrophysiology, with *CACNA1H* being particularly implicated postnatally [42,43].

We also demonstrate that our experimental group has higher expression of a range of metabolic genes, including the important transcription factor *PPARGC1A,* which drives mitochondrial biogenesis and oxidative metabolism [44]. Furthermore, we also observe differences in genes coding for adrenergic signalling-associated proteins between our Gremlin 2/RA-treated experimental group and the control, particularly across regulatory cAMP/cGMP phosphodiesterase proteins. Finally, we observe robust expression of *GJA1*, and *ATP1A1* across our experimental and control group, and slightly increased *GJA5* expression in our experimental group—suggesting that our experimental group retains expression of expected genes coding for sodium conductance-related proteins, while moving towards an atrial phenotype.

We sought to focus on several key genes to further validate these findings. First, we show the expression distribution spread, using our scRNASeq results, showing increased expression across the population of our experimental group for *MYL7, KCNJ3, NPPA* and *GJA5* (figure 2*f*). Then we sought to validate these findings using an orthogonal method: quantitative polymerase chain reaction (qPCR). *NPPA* expression was examined over the first 20 days, while expression for the other genes of interest was measured on day 15 (figure 2*h*). In agreement with our scRNASeq results, qPCR showed significantly higher expression of the atrial-specific genes *NPPA, MLY7,* and preferentially expressed atrial genes such as *KCNJ2, KCNA5* and *PITX2* in cells from the Gremlin 2/RA treatment group. However, the control group had much higher expression of ventricular-associated genes such as *MYL2* and the transcription factor *IRX4* [45] Of particular note, the expression of *MYL7*, the gene encoding myosin light chain 2 atrial isoform (*MLC2A*), is 20-fold higher in cells from the experimental Gremlin 2/RA treatment group than in hiPSC-derived CMs differentiated by the control commercial protocol, which is consistent with abundance of *MYL7* in adult atrial CMs [36]. Therefore, we validated the distinct atrial-like transcriptomic phenotype observed in our Gremlin 2/RA-treated experimental group.

### (c) Electrophysiological characterization reveals Gremlin 2/retinoic acid treatment produces a more atrial-like electrophysiological behaviour

**Figure 3.**
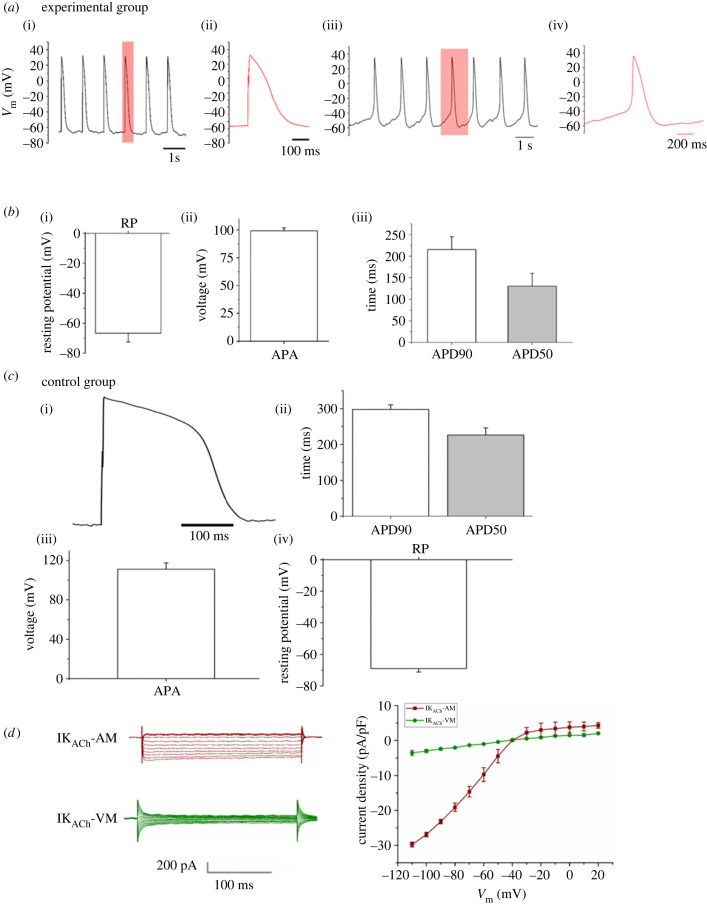
Electrophysiological characterization. (*a*) AP traces recorded from quiescent (i,ii) and spontaneous beating (iii,iv) cardiomyocytes (CMs) in the experimental group with an atrial-type morphology. (*b*) Mean values shown as bar graphs for (i) resting membrane potential (RP), (ii) APA and (iii) APD at 90% repolarization (APD_90_) and 50% repolarization (APD_50_), measured from seven cells prepared using the experimental protocol. (*c*) Records from cells prepared using the control protocol and having a ventricular phenotype showing (i) a representative AP trace, (ii) mean APD_90_ and APD_50_, (iii) APA and (iv) RP measured from five control hiPSC-derived CMs. Data are presented as mean ± s.e.m. (*d*) Currents induced by exposure to acetylcholine (ACh; 1 µM) under whole-cell voltage clamp conditions for voltage steps in the range −110 to +20 mV recorded from an example cell in the experimental group with an atrial morphology are shown as red traces and labelled as I_KACh_-AM. Corresponding traces from a control cell with a ventricular phenotype showing little or no current induced by ACh are shown in green and labelled as I_KACh_-VM. The I–V curve showing a plot of current density against membrane potential (V) is shown in the right panel. The mean currents induced by ACh in cells prepared with the experimental protocol and having an atrial morphology are shown in red. The curve shows the expected inward rectification with a steep increase in current at negative potentials as compared with the flatter slope at depolarized potentials. There was little or no current in cells from the control group with a ventricular phenotype (green).

The electrophysiological properties of atrial CMs are distinct from those of ventricular CMs. The resting membrane potential for human AMs has been reported around −74 mV, which is more depolarized than in ventricular cells (*ca* −81 mV) [30]. Additionally, APs in atrial cells have a smaller upstroke amplitude with the absence of a prominent plateau phase during the repolarization process [30]. Observations in animal models reproduce these findings that atrial cells have smaller amplitude and shorter duration APs [46]. Electrophysiological characteristics were investigated in our Gremlin 2/RA-treated hiPSC-derived atrial CMs on day 20–21 of differentiation and compared with the observations in adult human atrial and ventricular myocytes described above. The cultures were dispersed into individual myocytes as described for the immunohistochemistry experiments reported (see Material and Methods). AP recordings of the experimental group cells were obtained by patch clamp under the whole-cell configuration. Patch clamp results revealed an atrial-like AP in the experimental group cells as shown in figure 3*a*. Spontaneous activity was also detected in a small subset of cells (figure 3*a*(iii,iv)) while most others were quiescent until stimulated to fire APs by application of current stimuli through the patch electrode (figure 3*a*(i,ii)). Particularly, atrial characteristics were the absence of a prolonged plateau and relatively rapid repolarization. The action potential duration (APD) at 90 and 50% repolarization (APD_90,_ APD_50_) observed in seven experimental cells were 215 ± 30 ms and 130 ± 30 ms (*n* = 7). The average resting membrane potential of examined hiPSC-derived atrial CMs was −67 ± 6 mV (*n* = 7), which is close to that obtained from human atrial CMs −74 mV as previously reported [30]. In addition, action potential amplitude (APA) was 99 ± 3 mV recorded in our hiPSC-derived atrial CMs. This is comparable to that of human adult atrial CMs which is 89 ± 11 mV [30]. The observations on resting potential, APA and APD are shown as bar graphs in figure 3*b*.

By contrast, the morphology and characteristics of the APs recorded from our control commercially treated cells show ventricular-like features as shown in figure 3*c*. For example, the average resting membrane potential of commercially derived hiPSC-CMs was −68 ± 2 mV (*n* = 5). The APD_90_ and APD_50_ observed in these same control group cells were 297 ± 13 ms and 226 ± 20 ms (*n* = 5). In addition, APA was 111 ± 6 mV recorded in these control iPSC-CMs (*n* = 5).

To further examine the characteristics of the Gremlin 2/RA-treated hiPSC-derived atrial CMs: we assayed their response to acetylcholine (ACh) through the key atrial ACh-activated inwardly rectifying potassium current I_KACh_ [47,48]. Figure 3*d* shows currents induced by exposure to ACh under voltage clamp conditions. The panel on the right in figure 3*d* shows a plot of current against voltage. The ACh-induced current showed the expected inward rectification behaviour with a steeper slope of current against voltage for inward currents at more negative potentials as compared to the flatter curve for outward currents at depolarized membrane potentials. By contrast, cells from the control group of hiPSC-CMs, with a more ventricular phenotype, showed little or no current in response to ACh (figure 3*d*). The observations showing I_KACh_ in hiPSC-derived atrial CMs provide a functional counterpart to the observations in figure 2 showing robust expression of *KCNJ3* that encodes the protein GIRK1, that in turn comprises the channel which carries I_KACh_ [48].

In summary, the characteristics of APs recorded from our Gremlin 2/RA-treated hiPSC-derived atrial CMs were broadly similar to those reported for adult human atrial CMs and demonstrated differences from the control group consistent with the difference from more ventricular cells.

### (d) *α*- and *β*-adrenergic stimulation increased the calcium transient amplitude in our Gremlin 2/retinoic acid-treated human-induced pluripotent stem cell-derived atrial cardiomyocytes

**Figure 4.**
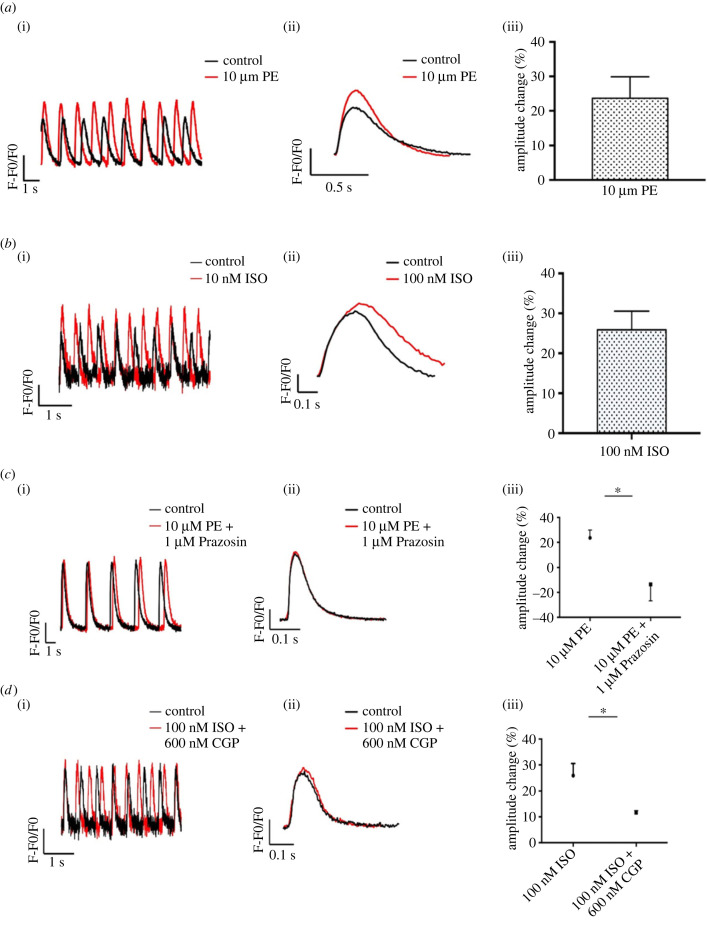
Changes in Ca^2+^ transient (CaT) amplitude in response to *α-* and *β-*adrenoceptor agonists in experimental group iPSC-CM cultures treated with Gremlin 2/RA. (*a*) CaTs (measured using Fluo-4 as the Ca^2+^ probe) in one representative experiment before and after 10 min of exposure to the *α*-adrenoceptor agonist, PE (10 µM). In this and following panels, raw traces are shown in (i), averaged traces are shown in (ii), and graphs representing the mean increase in CaT amplitude are shown in (iii). Red traces in (i) and (ii) are in the presence of drugs while black traces are in their absence. A bar graph showing the mean increase in CaT in response to PE is shown in (iii). (*b*) CaTs in a representative experiment before and after 10 min of exposure to the *β-*adrenoceptor agonist isoproterenol (ISO; 100 nM). A bar graph showing the mean increase in CaT in response to isoproterenol is shown in (iii). (*c*) CaTs in response to PE (10 µM) in the presence of the *α*-adrenergic antagonist (1 µM prazosin). CaTs after 10 min exposure to PE were abolished by pretreatment with prazosin. Graph showing the mean changes in CaT amplitude in response to PE in the absence and presence of prazosin is shown in (iii). (*d*) CaTs in response to ISO (100 nM) in the presence of the *β*1-adrenergic antagonist (300 nM GCP20712A). CaTs after 10 min exposure to ISO were abolished in iPSCs pre-treated with GCP20712A. Graph showing the mean changes in CaT amplitude in response to isoproterenol in the absence and presence of GCP20712A is shown in (iii). Four independent experiments were conducted for each drug combination shown in (*a*)–(*d*) (ii) and (iii). Data in (*a*)–(*d*) (iii) are shown as mean ± s.e.m., *p < 0.05.

We also tested whether our Gremlin 2/RA-treated hiPSC-derived atrial CMs responded to adrenoceptor stimulation. The presence of a functional *α*_1_-adrenoceptor signalling pathway was first tested by exposure of hiPSC-derived atrial CMs to phenylephrine (PE) (an *α*-adrenoreceptor agonist), which in adult atrial CMs increases Ca^2+^ currents [49]. hiPSC-derived atrial CMs from days 25 to 30 were used to assess the effect of PE on Ca^2+^ transient amplitude. Figure 4*a* shows that 10 µM PE increased Ca^2+^ transient amplitude by 28 ± 10% as measured from the fluo-4 fluorescence (*p* < 0.05, *n* = 5 batches of cells) but had no effect on the Ca^2+^ transient rise time or decay time. The involvement of *α*-adrenoceptors was further evaluated by 1 µM prazosin, a selective a_1_-adrenoceptor antagonist, which by itself had no effect on the amplitude of Ca^2+^ transients (*p* < 0.05, *n* = 4 batches of cells, data not shown). Figure 4*c* shows that in the presence of 1 µM prazosin, 10 µM PE did not cause any significant change in the amplitude of Ca^2+^ transients (*p* > 0.05, *n* = 4 batches of cells), consistent with the blockade of a-adrenoceptors by prazosin and the consequent suppression of the action of the agonist PE under these conditions (*p* < 0.05, *n* = 4 batches of cells).

*α*- and *β*-adrenergic receptors are highly expressed in human heart, with *β*-receptors more abundant than *α*-receptors. *β*-receptor stimulation leads to increased synthesis of cAMP and activation of protein kinase A which subsequently phosphorylates L-type Ca^2+^ channels and phospholamban, leading to increased Ca^2+^ influx and Ca^2+^ uptake into the SR [50]. As shown in figure 4*b*, 10 min of 100 nM isoprenaline (ISO) (a non-selective *β* agonist) treatment led to an increase of 26 ± 5% in the calcium transient amplitude (*p* < 0.05, *n* = 4 batches of cells). As shown in figure 4*d*, the involvement of *β*1-adrenoceptors in this response was tested by exposure of the cells to a selective *β*1 antagonist: CGP20712A (600 nM). When CGP20712A was present, 100 nM ISO had no significant effect on the amplitude of Ca^2+^ transients (*p* > 0.05, *n* = 4 batches of cells). It therefore appears that both *α* and *β* adrenoceptors are present and functional in our Gremlin 2/RA-treated hiPSC-derived atrial CMs, although we did not detect any changes in calcium removal back in the SR, which were expected (table 1).

**Table 1.** xx.

protocol	structure	electrophysiology	autonomic pharmacology	molecular markers
native human atria				
Benardeau, Amos, Dobrev, Grandi, Pandit, Wang, Jeevaratnam, Bosch, Bolger, Cui, Li, [20–29]	CMs demonstrate an elongated rod-like morphology, with coordinated and striated myofibrillar ultrastructure. Single nucleus [20]	CMs demonstrate a more triangular-like AP morphology that is also shorter than the ventricular APD. APD_90_ approximately 150–500 ms, APD_50_ approximately 8–150ms, APD_30_ approximately 4–24 ms. CMs have a more depolarized resting membrane potential (approx. −74 mV) than their ventricular cells (approx. −81 mV) [21–25,30]	respond to adrenergic stimuli with positive inotropic and chronotropic effects, which are opposed by cholinergic stimuli. Respond to a range of neurohormonal stimuli at nM concentrations. (e.g. 1 nM ISO). Respond to parasympathetic stimulation: activation of I_KACh_ in response to 1 µM ACh (−20 pA/pF at −90 mV) [26,27]	differ at the transcriptomic level from both the ventricles and more immature atria. A ‘master’ transcription factor (NR2F2) activates and maintains the atrial transcription program which differs both from the ventricular programme, and from that of the immature atrium across a range of structural and functional gene sets [28,29,31]
RA				
Devalla [8]	beating embryoid bodies from hESC, with mostly roundish cells; few with clear organized myofilaments	AP with pronounced phase 1 repolarization. Reduced AP plateau and APA_max_ height compared to control. Shorter APD_20_ (20.8 ± 3.7 ms), of ventricular APD_50_ (44 ± 10 ms), and APD_90_ (145 ± 21 ms) compared to control. No difference in RMP (*ca* −72 mV) from control	10 µM CCh induced a CCh-sensitive current (I_KACh_) with −2 pA/pF at −120 mV	downregulation of ventricular markers such as IRX4, MYL2 and HEY2 with concomittant upregulation of atrial markers such as NPPA, SLN, PITX2, NR2F2 and KCNA5. Demonstrated a COUP-TFII-dependent atrial transcriptome. Increased expression of atrial-enriched potassium channel subunit-encoding genes
Li [31]	formed cell sheets and EHT with organized sarcomeric structure	fired triangular-like APs, with a mean diastolic potential of −77.4 ± 1.4 mV, with a shorter APD than the ventricular control, but the same as the sinoatrial control. However, atrial iPSC had a higher maximal upstroke velocity than the sinoatrial group	responded to noradrenaline (100 nM) to reduce cycle length as single cells, and dose-dependent increase in beating rate of atrial EHT from hiPSC. 1 µM CCh increased single-cell iPSC-AM spontaneous beating cycle length, this occurred in a dose-dependent manner in atrial engineered heart tissue (EHT)	increased expression of *NPPA, NR2F2* and *KCNJ3* compared to ventricular controls, but only *NPPA* was higher compared to the sinoatrial controls
Cyganek [12]	formed cell sheets with good alignment of myofilaments in some areas	fired triangular-like APs (APD_20_/APD_80_ of 0.12 ± 0.01), with an APD_50_ of 167 ± 5 ms. Shorter more triangular calcium transients than control	positive inotropic response to 1 µM ISO. Negative inotropic response to 10 µM CCh	comprehensive increase in atrial markers such as *NPP A, NR2F2, KCNJ3, HOXA3, KCNA5,* along with a decrease in ventricular markers such as *MYL2, IRX4, MYH7, LPL* and *HEY2*. Confirmed the proteomic signal of atrial markers
Argenziano [11]	fibre-like tissue structures form from cell sheets of hiPSC-AMs	fired triangular-like APs (APD_90_/APD_50_ 1.3 ± 0.01), from a RMP of −45.2 ± 2.7 mV, with an APD_50_ of 57.5 ± 2.7 ms, an APD_90_ 89 ± 4.5 ms	10μM CCh activated I_KACh_ of −2.5 pA/pF at −120 mV. 100 µM adenosine also activated an I_KACh_ current of −1 pA/pF at −120 mV	downregulated *MYH7*, *MYL2* and MYL2 at the protein level as MLC2v but upregulated *KCNJ3,* and *KCNA5, NR2F2,* GJA5 and KCNA5, COUP-TFII, Ca_V3.1_ at the protein-level
Laksman [10]	elongated cells in sheets with organized sarcomeric striations along the long axis	fired APs with a pronounced notch and plateau. APs arose from a RMP of −56 ± 2 mV, with an APD_30_ of 37 ± 11 ms and APD_90_ of 247 ± 37 ms. Atrial sheets can produce organized rotors	not shown	upregulated *NPPA, KCNJ3* and *GJA5.* downregulated MYL2 and IRX4
Pei [32]	formed beating cell sheets, with striated sarcomeric ultrastructure	fired triangular APs, with an APD_90_ of *ca* 75 ms, from a mean diastolic potential of *ca* −55 mV	not shown	downregulated *IRX4*, and upregulated *NR2F2* compared to control. Atrial cell sheets were also MLC2v negative
Goldfracht [33]	organized into ring-shaped EHT	fired triangular APs, with an APD_90_ 205 ± 9 ms, APD_50_ 147 ± 10 ms, and APD_30_ 115 ± 9 ms from a RMP of 63 ± 2 mV	10 µM ISO increased force of contraction by *ca* 70%. Responded to CCh in a dose-dependent manner (2.5, 5, 10 µM), with a reduction in APD_90_ from 297 ± 16 ms at 0 µM, to 233 ± 17 ms at 10 µM CCh	lower MLC2v expression than ventricular control. Atrial EHT upregulated *KCNA5, KCNJ3, NPPA, MYL7, NR2F2* and downregulated *MYL2, MYH7, HEY2*. Atrial EHT also had higher SLN and CX40 expression
GREM2				
Tanwar [19]	not shown	CGR8 mESCs-CMs fired triangular APs, with an APD_90_ of approximately 40 ms versus control of approximately 70 ms which have a pronounced notch and dome morphology	not shown	CGR8 mESCs-CMs had higher expression of Nkx2.5, and Myh6. GREM2 treatment also increased expression of Tnnt2, Gata4, Myl7, Gja5, Kcnj5 and Hey1 compared to control. Whereas, GREM2 treatment decreased expression of more ventricular transcripts such as *Myl2, Gja1* and *Kcnq1*
Bylund [34]	increased % contracting cells, and increased number of CPCs	not shown	not shown	increases expression of *NKX2.5*, *MYH6*, *TNNT2* and *MYL7*. GREM2 effect was JNK-dependent
GREM2 and RA				
Ahmad (this paper)	elongated rod-like morphology in some, with organized striated sarcomeres. iPSC-AMs form cTnT^+^ beating cell sheets	fired triangular-like APs, although with some plateau, from a baseline RMP of −67 ± 6 mV. APs have an APD_90_ 215 ± 30 and APD_50_ 130 ± 30 (APD_90_/APD_50_ *ca* 1.7)	responsed to *α*- (10 µM PE) and *β*-adrenergic (100 nM ISO) stimuli, sensitive respectively to blockade by prazosin (1 µM *α*-antagonist) and CGP20712A (600 nM, *β*_1_-selective antagonist). *α*- and *β*-stimuli both increased calcium transient amplitude. Demonstrated I_KACh_ current *ca* –30 pA/pF at −110 mV with 1 µM ACh	increased COUP-TFII and NPPA expression relative to control. At the mRNA level, expressed a range of cardiac TFs, including NR2F2, coupled to downstream-effector modules such as GATA4, GATA5, GATA6, HEY1, SFRP5 and MEF2D. higher expression of a range of atrial-associated transcripts compared to control (e.g. NPPA, MYL7, KCNA5). Robust expression of contractile and calcium-handling proteins

## 3. Discussion

In the report presented here, we have developed a new protocol to generate functionally responsive atrial CMs from human iPSCs. The protocol involves temporal regulation of WNT and BMP signalling and the resultant cells structurally and functionally resemble atrial CMs in terms of atrial transcription programmes, electrophysiology and morphology.

The key findings in this report are: (i) development and validation of a new protocol consisting of treatment with Gremlin 2 and RA that permits differentiation of CMs with an atrial phenotype from human iPSCs; (ii) a substantial fraction of cells generated by this protocol showed a rod-shaped morphology with a single central nucleus similar to that observed in cells isolated from human atria; (iii) these myocytes demonstrated a distinctly atrial transcriptional programme; (iv) most isolated rod-shaped cells were electrically quiescent until stimulated to fire typical APs with an amplitude of 100 mV arising from a resting potential of approximately −70 mV; and (v) hiPSC-derived atrial CMs from this protocol responded to adrenergic stimuli by both *α*- or *β*-adrenoceptor agonists, and to cholinergic stimuli.

Development of our Gremlin 2 and RA combination treatment protocol for iPSC-AMs differentiation was based on previous observations showing that both of these agents can separately influence heart development (table 1). In zebrafish, Gremlin 2 was essential for the development of the heart [18]. Muller *et al*. [18] also showed that the ventricular and atrial CM specification was affected by the expression of Gremlin 2 because of its role in mediating phosphorylated SMAD1/5/8 and BMP signalling. Additional work [19] showed that Gremlin 2 enhanced cardiac differentiation and increased the atrial cell population in mouse ESC-derived CMs, as was evident from electrophysiological studies demonstrating a mixture of different AP shapes (ventricular, atrial and nodal) in untreated cells, while a dominant atrial-type AP was recorded from Gremlin 2-treated cells. Gene expression analysis also showed significantly higher expression of atrial-specific genes, *MYL7*, *GJA5*, *KCNJ5*, *CACNA1D1*, *SLN* and *NPPA* in the cells receiving Gremlin 2 treatment [15]. RA has separately been shown to influence heart development, by actions that are thought to involve modification of BMP signalling [51]. Currently, a range of RA-based protocols exist for iPSC/ESC differentiation into atrial CMs and similarly for Gremlin 2 [8–10,12,13,19,33,34,52,53] (table 1). While these differentiation protocols have shown success in establishing an atrial-like phenotype, they mostly produced immature cells, particularly in terms of electrophysiology and pharmacology. Thus, while the two agents have been shown separately to reliably induce an atrial-like phenotype, we sought to understand their combinatorial effect. It is interesting to speculate on the precise mechanism through which Gremlin 2 and RA synergize, as previously attempts with Noggin and RA have also produced atrial-specific CMs, albeit with a less robust atrial phenotype [54]. Both Noggin and Gremlin 2 both possess BMP antagonism [19]. However, Gremlin 2 activates *COUP-TFII* and *HEY1* in a JNK-sensitive manner not observed with Noggin signalling [19]. It is beyond the scope of this study, but it will be interesting to further characterize the putative role of JNK signalling in atrial differentiation. The combined use of Gremlin 2 and RA introduced in our experiments described above substantially increased the effectiveness of the new protocol in directing iPSC development towards a more mature atrial phenotype and excitingly across several structural and functional domains of maturity.

An important feature of the cardiac cells produced by the novel methods described here is a morphology that resembles the adult atrial phenotype. The cells had a single nucleus and approximately 40% showed an elongated ‘rod’ shape that is characteristic of adult myocytes. This was particularly evident in isolated cells that had been dispersed from cell cultures (figure 1*c*). Immunohistochemistry demonstrated that the expression of atrial-specific proteins, particularly COUP-TFII and NPPA, was greater in cultures from the Gremlin 2/RA-treated experimental group than in cultures from the commercially derived group (with 78% CMs showing COUP-TFII expression in the treated experimental group as compared with 9% in the control group).

A more extensive transcriptome analysis by single-cell RNA-sequencing on over 300 cells from each group provided further evidence that Gremlin 2/RA treatment elicited a more atrial transcriptional programme than in the control group: atrial genes showing enhanced expression included: *MYL7, HEY1, HOXA3, SLN, MYH6, KCNH2, GATA4 and NPPA*; whereas the control group had a predominately more ventricular transcriptomic signature, with greater expression of ventricular transcription factors such as *IRX5* and *IRX4.* We used the unbiased nature of scRNAseq to cross-validate our protein-level expression of atrial markers following Gremlin 2/RA treatment (figure 1). We observed the Gremlin 2/RA-treatment produced an increased percentage of atrial CMs from 16% in the control group to 71% in the experimental group. Particular genes that were upregulated were those that are important for atrial cell function, including ion channels (e.g. *KCNA5, KCNJ2, KCNJ3* and *KCNH2*) and calcium-handling proteins (e.g. *RYR2, CACNA1H, CACNA1G* and *SLC8A1*). It is interesting to note that our scRNAseq analysis suggests that despite robust expression of *NR2F2* in the control group to a somewhat comparable degree as the experimental group (figure 2*d*), this does not appear to be coupled to an increase in the protein levels of its gene product COUP-TFII (figure 1*b,d*), nor to an atrial transcriptional programme (figure 2). This highlights the opportunity of *in vitro* differentiation protocols to study normal developmental signalling and mandates further study to ascertain the epigenetic regulation of *NR2F2*.

In view of the above messenger RNA (mRNA) expression of genes coding for atrial ion channels, it is perhaps unsurprising that electrophysiological recording provided important additional information supporting the effectiveness of the new Gremlin 2/RA treatment in directing iPSC development towards the adult atrial phenotype. When single cells were isolated from the cultures in which they were grown, they were generally electrically quiescent, and brief current stimuli were needed to initiate APs, to the authors' knowledge this has not been previously reported. The resting membrane potential approached −70 mV, and the amplitude of APs was approximately 100 mV consistent with an overshoot of approximately 30 mV. The shape and duration of APs was also broadly consistent with the adult atrial phenotype. Similarly, the amplitude of APs using the improved protocol with a combination of Gremlin 2 and RA is also larger than in previous reports using either as a differential factor in isolation [9,33,52,54]. In particular, it is important to note the improved activity and larger current we recorded from the preferentially atrial current I_KACh_ compared to previous RA-only protocols [8,13], with ours much closer to that seen in adult human atrial CMs [55]. Additionally, our robust I_KACh_ signal was concomitant with more clear mature sarcomeric subcellular structure, which was less obvious in other protocols with robust I_KACh_ signal [8]. We highlight I_KAch_ for particular note given the major importance of this current in atrial repolarization and membrane stability and its implication in AF [56,57] which makes a robust I_KACh_ current probably a necessity for meaningful modelling of AF, drug development and translational work using hiPSC-derived atrial CMs. Although the above characteristics show close similarities to those of adult CMs, there is always the prospect of making these similarities closer still and a recent review [58] discusses variability and disparity of hiPSC characteristics across different ‘-omic’ levels, while recognizing the importance of these approaches in characterizing PSC-derived CMs. Further study interrogating more detailed electrophysiological behaviour of our iPSC-AMs will help elucidate the complexities. Nevertheless all the features discussed above make iPSC-AMs derived using our new Gremlin 2/RA protocol suitable for the testing of beneficial and harmful effects of drugs on human cardiac atrial muscle.

We also sought to investigate the dominant electrophysiological properties of the two cell culture conditions (experimental against control) beyond individual currents. While it would have been interesting to see the difference between our hiPSC-derived atrial CMs, and atrial CMs from the control group treated with commercial reagents only, we compared the dominant and modal phenotype in each condition. This ventricular-like and atrial-like behaviour from the control and experimental groups, respectively, was representative of all of the cells we assayed in either group via patch clamp. The modal cell in a given culture will influence the pre-dominant electrophysiological phenotype, and thus our results give an unbiased consideration of the two groups, without any bias introduced through positive or negative selection of the cells for comparison.

Another important characteristic of the iPSC-AMs produced by this protocol that provides a useful experimental model for studying human cardiac muscle function is the ability to respond to selective neurohormonal signalling agonists: PE, ISO and ACh. The observation that the adrenergic effects could be blocked by the appropriate antagonists provided further support for the expression and functional intracellular coupling of both *α* and *β* adrenoceptors in the cells prepared by the methods described here (figure 4). The changes in the Ca^2+^ transient amplitudes that were observed in response to both *α* and *β* adrenoceptor stimulation are consistent with the presence and activity of the intracellular cAMP signalling modules downstream of adrenoreceptor stimulation. We did not detect a clear positive lusitropic effect from *β* adrenoceptor stimulation, but this point will be the subject of more detailed future studies. However, to the knowledge of the authors, this is the first iPSC-derived atrial CM differentiation protocol to produce a robust response to adrenergic stimuli without further maturation protocols [8–15]. Furthermore, our I_K__ACh_ current density in response to 1 µM ACh was equivalent to native human mature atrial CMs. The ability of our iPSC-AMs to respond to both adrenergic and cholinergic activators provides a useful model for studying the interaction of these key cardiac modulators in atrial disease.

In summary, the key features of the cells that mark out advances of the approach presented are rod-shaped morphology in a large fraction of the cells (approximately 40%); expression of atrial-specific transcripts; electrophysiological charactersistics, closely resembling the adult atrial phenotype; and ability to respond to adrenoceptor and cholinoreceptor stimulation. It seems likely that these human cells, with characteristics closely resembling those of the adult atrial phenotype, will provide an important resource for drug testing (particularly drugs to treat AF) and for the investigation of cell signalling mechanisms.

## 4. Material and methods

### (a) I nduced pluripotent stem cell culturing, differentiation and characterisations

The iPSCs lines used for the CM differentiation were purchased from Gibco® Life Technology, Carslbad, USA or developed in the laboratory. Cord blood-derived CD34+ progenitor cells were reprogrammed into iPSCs by using a three-plasmid and seven-factor episomal system (OCT4, Sox2, Myc, Klf4, Nanog, SV40LT and Lin28 antigen). iPSC lines were fully characterized by (i) immunostaining, (ii) qPCR, (iii) tri-lineage differentiation, (iv) teratoma essay and (v) karyotyping as described in Appendix.

iPSC line was cultured on a Matrigel-coated six-well culture plate with mTeSR™1 medium (STEMCELL™ Technologies), human iPSCs were maintained undifferentiated with daily medium change as the protocol described in detail in Appendix.

### (b) Cardiomyocyte isolation and immunofluorescent characterization

CM aggregates were dissociated by using Accutase™ (STEMCELL™ Technologies). Following a Dulbecco's phosphate buffered saline wash, Accutase™ was added to the CMs for 8 min at 37°C. After the addition of 2–3 volume of fresh cardiomyocyte maintenance medium (CMM) to terminate the reaction of Accutase™, the cells were firstly physically detached and collected by 1000 µl pipette. Subsequently, dissociated CMs were transferred and seeded to a 1 : 30 Matrigel-coated polymer-made coverslip. They were cultured with CMM and 10 µm Y-27 683 for at least two days prior to immunofluorescent examination. The dissociated iPSC-derived CMs cultured on the polymer-made coverslips (ibidi Technologies) were washed with PBS and fixed with 4% paraformaldehyde [17] at room temperature for 20 min and then went through various steps of staining as described in Appendix in detail. The immunocytochemistry images were analysed by ImageJ.

### (c) Single-cell RNA-sequencing and RT-qPCR

Human iPSC-derived CMs were collected as the protocols described in Appendix and were fixed by chilled methanol and stored on ice for 15 min for fixation prior to −80°C storage. The fixed cells were rehydrated using a FACS Aria II or FACSJazz (BD Biosciences). Single cells (based on DAPI exclusion and forward/side scatter properties) were sorted into 384-well hard-shell plates (Biorad) with 5 µl of vapour-lock (QIAGEN) containing 100–200 nl of RT primers, dNTPs and synthetic mRNA Spike-Ins and immediately spun down and frozen to −80°C. Sort-sequencing was used for single-cell RNAseq. In brief, CMs were lysed by 5 min at 65°C, when RT and second strand mixes were dispersed with the Nanodrop II liquid handling platform (GC biotech). The aqueous phase was split from the oil phase after pooling all CMs in one library, followed by IVT transcription. For library preparation, CEL-Seq2 protocol was applied. Primers were composed of a 4 bp random molecular barcode, 24 bp polyT stretch, a T7 promoter, a cell-specific 8 bp barcode and the 5′ Illumina TruSeq small RNA kit adaptor. Single-cell mRNA was subsequently reverse transcribed, converted to double-stranded cDNA, assembled and *in vitro* transcribed for linear as required for the CEL-Seq 2 protocol [59]. Illumina-sequencing libraries were then made with the TruSeq small RNA primers (Illumina) and sequenced paired-end at 75 bp read length the Illumina NextSeq. Read alignment was performed. Subsequently, candidate cells and genes were analysed in the R environment. Cells were filtered using a criteria in which transcript number > 12 000 and gene were filtered > 5 transcript in more than five cells. Differential gene expression analysis was performed via RaceID (v0.1.5) [60]. Violin plots were generated by using the R package ggplot2 (v3.2.1) [61] and heatmaps were made by using R package ComplexHeatmap (v2.1.2) [62]. GO enrichment analysis was performed and visualized using ClusterProfiler [63].

For RT-qPCR, various gene expressions were analysed to identify the derived cells as CMs, and to subsequently testify the subtype specificity of the derived CMs. Quantitative PCR analysis was conducted for atrial-specific genes (*NPPA*, *MYL7*), inward rectifier potassium channel coding genes (*KCNJ2*), other potassium channel-encoding genes (*KCNA5)* and ventricular-specific genes (*MYL2, IRX4).* All the TaqMan® Gene Expression Assays were predesigned by Applied Biosystems by Thermo Fisher Scientific. qPCR was performed with LightCyclerÒ480 (Roche) using TaqMan® Gene Expression Master Mix (Applied Biosystems by Thermo Fisher Scientific) under the instructions from the manufacturer.

### (d) Electrophysiological studies and Ca^2+^ imaging

AP recording was recorded by patch clamp under a whole-cell configuration using Axon 700B amplifier system (Molecular Devices, USA). For the records of APs, the pipette solution contained (in mmol l^−1^): K-aspartate 110, KCl 10, NaCl 5, MgCl_2_ 5.2, HEPES 5, K_2_ATP 5 and pH 7.2 with KOH. For the measurements of I_K__ACh_, the cells were voltage clamped and the pipette solution contained (in mmol l^−1^): K-aspartate 136, KCl 5.4, NaCl 5, MgCl_2_ 1, HEPES 1, EGTA 5, Mg-ATP 5, phosphocreatine 5, pH 7.2 with KOH. The bath solution consisted of (in mmol l^−1^) NaCl 136, KCl 5.4, MgCl_2_ 1.0, CaCl_2_ 1.8, NaH_2_PO4 0.33, HEPES 5, Glucose 10, pH 7.4 with NaOH. No compensation was applied for changes in junction potential during whole-cell recording, but correction with aspartate as the major anion would have resulted in a small shift in recorded potential in the hyperpolarizing direction [44]. In experiments where I_KACh_ was to be measured, cells were voltage clamped at a holding potential of −40 mV and step depolarizations were applied in the range −120 mV to +20 mV in increments of 10 mV. ACh was applied at a concentration of 1 µM and currents in the absence of ACh were subtracted from currents in the presence of ACh to give I_KACh_.

Optical mapping experiments were carried out after the cells started to contract. The responsiveness of the derived CMs in the experimental group to *β*- and α-adrenergic receptor agonists and antagonists was measured. Intracellular calcium transients were analysed using a 128 × 128 EMCCD camera (Photometrics, Tucson, USA). The derived CMs were pre-loaded with 1 µM Fluo4 (Molecular Probes by Life Technologies) dissolved in dimethyl sulfoxide for 15 min at 37°C in CMM. Calcium transients of the derived CMs were first recorded without the addition of the drugs as control. Recordings were then taken at 0 min, 5 min, 10 min and 15 min after 100 nM ISO treatment. Using the same method, the responsiveness of the derived CMs in the experimental group to 10 µM PE treatment was tested. In adrenergic receptor antagonist tests, the cells were pre-treated with 600 nM CGP20712A (CGP) (Sigma) dissolved in Fluo4-loading solution prior to 100 nM ISO treatment. Recordings were taken in the same way as described above. Similarly, the cells were pre-treated with 1 µM prazosin dissolved in Fluo4-loading solution before a treatment of 10 µM PE. Metamorph was used to take the recordings, and 4000 or 8000 frames were taken for each recording, with a frame rate of 100 Hz (for 4000 frames) or 333.33 Hz (for 8000 frames). Regions of interest were selected using ImageJ. Raw traces were analysed and baseline corrections were conducted using Clampfit. Calcium transient amplitude change was calculated based on averaged trace. Electrophysiological analysis was performed, in part, using ElectroMap [64] on MATLAB. To calculate beat-to-beat CaT50, the mean signal was segmented into individual beats. Representative regions were defined as two 16 × 16 pixel regions.

## Data Availability

All data are contained within the manuscript. Reasonable requests for raw data and analysis scripts should be made to the corresponding author.
